# Clinical and Biochemical Effects of Antioxidant Gel as a Local Drug Delivery Agent in Stage II Grade A Periodontitis Patients: A Prospective Clinical Study

**DOI:** 10.7759/cureus.61707

**Published:** 2024-06-05

**Authors:** Nivedha Nedumaran, Arvina Rajasekar

**Affiliations:** 1 Department of Periodontology, Saveetha Dental College and Hospitals, Saveetha Institute of Medical and Technical Sciences, Saveetha University, Chennai, IND

**Keywords:** local drug delivery agents, scaling and root planing, periodontitis, oxidative stress, antioxidant

## Abstract

Background

Periodontal tissue breakdown is mainly due to pathogenic bacteria and dysregulated immune response resulting in the production of reactive oxygen species/reactive nitrogen species (ROS/RNS) causing tissue degradation. Scaling and root planing (SRP) is usually done for the management of periodontitis. However, it has been reported that adjuncts like antibiotics, antiseptics, and antioxidants in the form of local drug delivery enhance the outcome of SRP.

Aim

The present clinical study aims to examine the efficacy of an antioxidant oral gel (Bluem®) as a local drug delivery agent adjunct to SRP in the management of stage II grade A periodontitis in terms of clinical and biochemical parameters.

Materials and methods

The prospective clinical study was conducted among 40 stage II grade A periodontitis patients. The participants were then divided into two groups: Group 1 (Control)-SRP alone (n=20) and Group 2 (Test)-antioxidant gel (Bluem®) with SRP (n=20). Clinical variables including plaque index (PI), gingival index (GI), probing depth (PD), and clinical attachment level (CAL) were recorded. Saliva (unstimulated) specimens were collected to measure total oxidant status (TOS), total antioxidant capacity (TAOC), and oxidative stress index (OSI). Specimen collection and assessment of clinical variables were done before intervention (baseline) and after three months. SPSS Software (Version 20.0, Armonk, NY, USA: IBM Corp) was used for statistical analysis. Intragroup and intergroup comparisons were done by paired t-test and independent t-test, respectively. A p-value <0.05 indicated that the result was statistically significant.

Results

On intragroup analysis, both the groups at three months revealed statistically significant improvement of PI, GI, PD, CAL, TOS, TAOC, and OSI (p<0.05) from baseline. Intergroup comparison in the third month showed a statistically significant improvement in favor of Group 2 in terms of all the clinical and biochemical parameters (p<0.05) except for PI (p>0.05).

Conclusion

The locally delivered antioxidant gel as an adjunct to SRP seems to be effective in reducing oxidative stress and improving the periodontal parameters among stage II grade A periodontitis patients.

## Introduction

Periodontitis is a multi-factorial oral disease that causes a progressive breakdown of the periodontal attachment [1]. A dysregulated host immune response, marked by an increase in the release of free radicals and inflammatory mediators, predisposes to the breakdown of periodontal tissue, where periodontopathic bacteria are required for the initiation of the disease. Pathogenesis involves neutrophil stimulation by periodontal pathogens, which results in a respiratory burst mechanism that in turn leads to the production of reactive oxygen species (ROS) such as superoxide anion radicals, hydroxyl radicals, hydrogen peroxide, and hypochlorous acid, which are directly accountable for the degradation of periodontal tissue extracellular matrix components such as collagen, elastin, proteoglycans, and glycosaminoglycans. As a result, the periodontal attachment is destroyed [2,3].

It has been established that ROS plays a specific role in the tissue degradation of periodontitis. When ROS are generated at high concentrations, they can lead to oxidative stress in tissues, which can directly harm cells and the extracellular matrix. Advanced glycation end products and lipid peroxide-modified proteins are examples of products of this oxidative damage that can cause more ROS-induced damage and neutrophil chemotactic activities [4]. The production and accumulation of antioxidants, which are materials that, if at low levels compared to that of an oxidizable substrate, considerably delay or prevent that substrate's oxidation. The activity of ROS is controlled and modulated by such antioxidants [5].

Scaling and root planing (SRP), which disrupts the biofilm, is a traditional therapeutic option for periodontitis [6]. However, the site of the lesion, tooth anatomy, and the virulence nature of the periodontal pathogens make periodontal therapy more difficult and hinder the reduction in the bacterial load. Therefore, in addition to SRP, systemic antibiotics were given. Systemic antibiotic therapy has some benefits, but it also has drawbacks, which include the development of resistance and systemic toxicity [7]. This led to the development of a local drug delivery system to achieve the necessary concentration of drugs at the target areas. Several locally delivered antimicrobial systems have been evaluated for their efficacy in the treatment of chronic periodontitis as an adjunct to traditional SRP [8]. Antioxidants have also been used in recent days at periodontitis sites to correct the oxidant-antioxidant imbalance, aiming to restore periodontal health [9].

Literature supports the administration of antioxidant mouthwashes and gels as a treatment for gingivitis and periodontitis [10]. However, there are no studies assessing the efficacy of antioxidant gel as a local drug in the management of periodontitis. In this context, the purpose of this study was to assess the effect of antioxidant oral gel (Bluem®) as a local drug delivery method in conjunction with SRP for the treatment of stage II grade A periodontitis in terms of clinical and biochemical parameters.

## Materials and methods

Study design

This prospective clinical study was performed between October 2022 and March 2023. Forty stage II grade A periodontitis patients in the age range of 20 to 55 years were chosen from Saveetha University’s Department of Periodontology. On clinical examination, a stage II grade A periodontitis diagnosis was made based on the classification of the American Academy of Periodontology, 2017 [11]. The study participants were divided into two groups: Group 1 (Control)-SRP alone (n=20) and Group 2 (Test)-antioxidant gel (Bluem®) with SRP (n=20) [12]. The study protocol approval was obtained from the Saveetha University Ethical Committee before the start of the study (IHEC/SDC/PERIO-2203/23/082). Every patient provided signed informed consent. The sample size was calculated using G*Power software version 3.1.9.4 (Heinrich-Heine-Universität Düsseldorf, Düsseldorf, Germany; power at 80% and alpha error at 95% confidence level) by considering the mean and standard deviation values from the earlier research [13].

**Figure 1:**
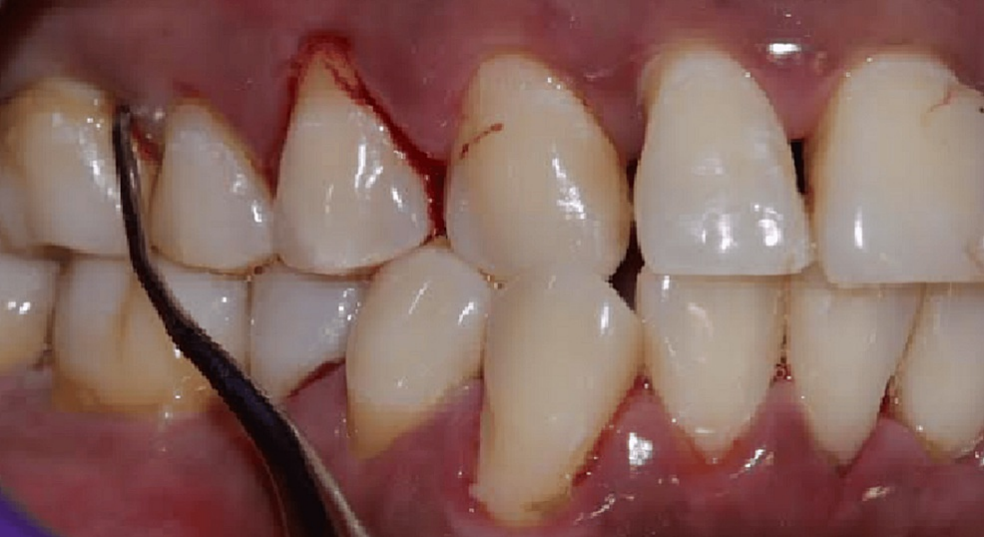
SRP done using sterile curettes (Hu-Friedy®, Chicago, US) SRP: scaling and root planing

Patients with no systemic disease; no periodontal treatment in the last six months of enrollment; no use of antibiotics, anti-inflammatory drugs, or antioxidants within three months of enrollment; no history of smoking; no history of diabetes or hypertension; no use of alcohol; age between 20 and 55; more than 18 teeth present; fewer than five decay cavities were enrolled in the study. Systemically compromised patients, pregnant and lactating women, patients under drugs or dietary supplements for six months before the intervention, and smokers were excluded from the study. Both the group patients underwent SRP (Figure 1) using sterile curettes (Hu-Friedy®, Chicago, US).

**Figure 2:**
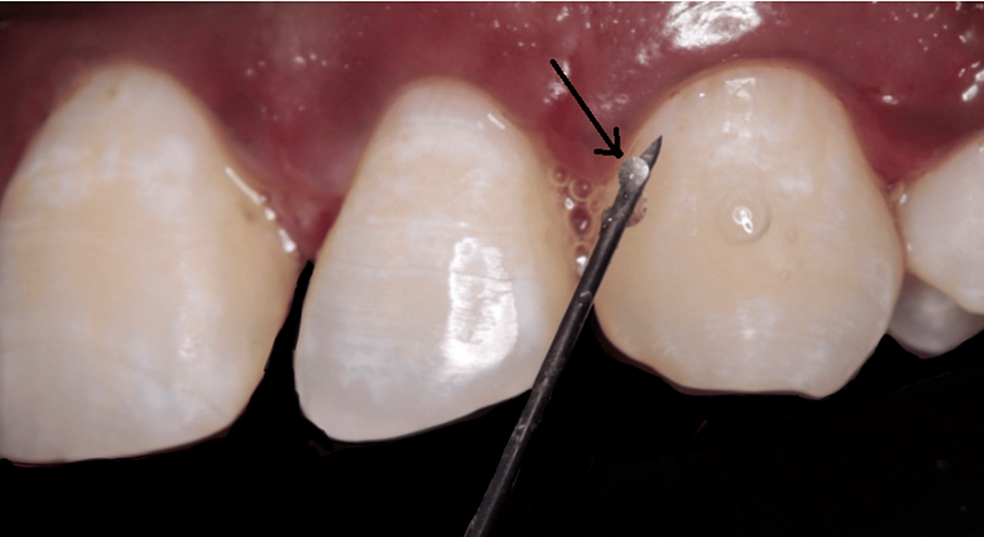
Bluem® gel application into the periodontal pockets using a syringe

After receiving SRP treatment, the test group participants had a single application of Bluem® gel into the periodontal pockets using a syringe with a 27 gauge needle (Figure 2).

Outcome parameters

Clinical variables such as plaque index (PI), gingival index (GI), probing depth (PD), and clinical attachment level (CAL) were noted. PI was recorded using Silness and Loe PI, where scores from four surfaces (distal, mesial, facial, and lingual) surrounding each tooth were calculated and the average was taken [14]. GI was recorded using Loe and Silness GI, where scores from four surfaces (distal, mesial, facial, and lingual) surrounding each tooth were calculated and the average was taken [14]. A University of North Carolina (UNC)-15 periodontal probe was used to record PD and CAL at six sites (mesio buccal, disto buccal, mid buccal, mesio lingual/palatal, disto lingual/palatal, and mid lingual/palatal) for each tooth, and the average was then calculated. All these measurements were performed before SRP (baseline) and after three months.

Between 8 am and 10 am, 5 mL of unstimulated saliva was collected from all the participants. Samples were collected and immediately centrifuged (5000×g, 20 minutes, 4°C). The supernatant fluid was utilized for further analysis. Each sample received 10 μL of 0.5M butylated hydroxytoluene (BHT)/1 mL of saliva as an antioxidant. The saliva was subsequently frozen at -80°C and subjected to biochemical analysis [13]. Biochemical variables comprising of total oxidant status (TOS), total antioxidant capacity (TAOC), and oxidative stress index (OSI) were measured. Saliva was defrosted at 4°C on the day of performing the assays. The colorimetric technique established by Erel et al. was used to calculate TOS [15]. Here, Fe^2+^ ions were converted into Fe^3+^ ions in the existence of oxidants included in the sample. After that, xylenol orange was used to detect Fe^3+^ ions. The hydrogen peroxide calibration curve was used to determine the TOS concentration. The determination of TOS was performed three times. The results were expressed as µmol H_2_O_2_ Equiv./L. The colorimetric technique established by Erel et al. was used to calculate TAOC [16]. This method's principle is based on measuring the ability of the antioxidant in the sample to neutralize 2,2-azino-bis-3-ethylbenzothiazoline-6-sulfonate (ABTS) cationic radical. At 660 nm, variation in the ABTS solution absorbance was observed. 5 μL of the sample was incubated at pH 5.8 with 200 μL of 0.4M acetate buffer to measure the TAOC content. Then, in 30 mm of acetate buffer at pH 3.6, 20 μL of ABTS solution was added. After the samples were incubated, the absorbance at 660 nm was determined. Trolox (6-hydroxy-2,5,7,8-tetramethyl-chroman-2-carboxylic acid) standard curve was applied to compute the TAOC concentration and reported as µm Trolox per mg of protein. The process of TAC determination was run three times. The ratio of TOS to TAOC was calculated to determine OSI and was expressed as a percentage [17]. Biochemical analysis was performed before SRP (baseline) and after three months.

Statistical analysis

Statistical analysis was done using the statistical package of social sciences (SPSS) software (Version 20.0, Armonk, NY, USA: IBM Corp). For intragroup and intergroup comparisons, the paired t-test and independent t-test were used, respectively. A p-value <0.05 indicated that the result was statistically significant.

## Results

Intragroup analysis following three months of SRP revealed statistically significant improvement from baseline in Group 1 with PI, GI, and PD reduced from 1.91 ± 0.61 to 0.16 ± 0.27, 1.41 ± 0.51 to 0.19 ± 0.51, and 4.30 ± 0.71 to 1.10 ± 0.50, respectively. CAL gain was observed from 4.70 ± 0.83 to 2.10 ± 0.51. TOS reduced from 9.07 ± 3.70 (baseline) to 6.13 ± 0.09 (three months). TAOC increased from 0.76 ± 0.31 to 1.81 ± 0.75, and OSI reduced from 1.25 ± 0.81 to 0.79 ± 0.80. All these differences were statistically significant with p<0.05 (Table 1).

**Table 1:** Comparison of clinical and biochemical parameters in Group 1 (Control) between baseline and three months *statistically significant PI: plaque index; GI: gingival index; PD: pocket depth; CAL: clinical attachment level; TOS: total oxidant status; TAOC: total antioxidant capacity; OSI: oxidative stress index

Variable	Pre-operative	Post-operative	P-value
PI	1.19 ± 0.61	0.16 ± 0.27	0.023*
GI	1.41 ± 0.51	0.19 ± 0.51	0.012*
PD (mm)	4.30 ± 0.71	1.10 ± 0.50	0.023*
CAL (mm)	4.7 ± 0.83	2.10 ± 0.51	0.011*
TOS (µmol H_2_O_2_ Equiv./L)	9.07 ± 3.70	6.13 ± 0.09	0.026*
TAOC (µm Trolox/mg)	0.76 ± 0.31	1.81 ± 0.75	0.032*
OSI (%)	1.25 ± 0.81	0.79 ± 0.80	0.017*

Intragroup analysis following three months of SRP revealed statistically significant improvement from baseline in Group 2 with PI, GI, and PD reduced from 1.32 ± 0.27 to 0.17 ± 0.32, 1.26 ± 0.50 to 0.13 ± 0.51, and 4.10 ± 0.86 to 0.50 ± 0.71, respectively. CAL gain was observed from 4.60 ± 0.51 to 1.50 ± 0.10. TOS reduced from 9.12 ± 2.60 to 3.63 ± 1.50. TAOC increased from 1.20 ± 0.11 to 2.60 ± 0.64, and OSI decreased from 1.34 ± 0.52 to 0.545 ± 0.37. All the differences were statistically significant with p<0.05 (Table 2).

**Table 2:** Comparison of clinical and biochemical parameters in Group 2 (Test) between baseline and three months *statistically significant PI: plaque index; GI: gingival index; PD: pocket depth; CAL: clinical attachment level; TOS: total oxidant status; TAOC: total antioxidant capacity; OSI: oxidative stress index

Variable	Pre-operative	Post-operative	P-value
PI	1.32 ± 0.27	0.17 ± 0.32	0.013*
GI	1.26 ± 0.5	0.13 ± 0.51	0.011*
PD (mm)	4.10 ± 0.86	0.50 ± 0.71	0.023*
CAL (mm)	4.60 ± 0.51	1.50 ± 0.10	0.017*
TOS (µmol H_2_O_2_ Equiv./L)	9.12 ± 2.60	3.63 ± 1.50	0.045*
TAOC (µm Trolox/mg)	1.20 ± 0.11	2.60 ± 0.64	0.023*
OSI (%)	1.34 ± 0.52	0.545 ± 0.37	0.029*

In the third month, the mean PI, GI, PD, CAL, TOS, TAOC, and OSI of Group 1 participants were 0.16 ± 0.27, 0.19 ± 0.51, 1.10 ± 0.50, 2.10 ± 0.51, 6.13 ± 0.09, 1.81 ± 0.75, and 0.79 ± 0.80, respectively. In the third month, the mean PI, GI, PD, CAL, TOS, TAOC, and OSI of Group 2 participants were 0.17 ± 0.32, 0.13 ± 0.51, 0.50 ± 0.71, 1.50 ± 0.10, 3.63 ± 1.50, 2.60 ± 0.64, and 0.545 ± 0.37, respectively. Intergroup comparison in the third month showed a statistically significant difference in favor of Group 2 in terms of all the clinical and biochemical parameters (p<0.05) except for PI (p>0.05) (Table 3). 

**Table 3:** Intergroup comparison of clinical and biochemical parameters at three months *statistically significant PI: plaque index; GI: gingival index; PD: pocket depth; CAL: clinical attachment level; TOS: total oxidant status; TAOC: total antioxidant capacity; OSI: oxidative stress index

Variable	Group 1 (Control)	Group 2 (Test)	P-value
PI	0.16 ± 0.27	0.17 ± 0.32	0.06
GI	0.19 ± 0.51	0.13 ± 0.51	0.03*
PD (mm)	1.10 ± 0.50	0.50 ± 0.71	0.04*
CAL (mm)	2.10 ± 0.51	1.50 ± 0.10	0.03*
TOS (µmol H_2_O_2_ Equiv./L)	6.13 ± 0.09	3.63 ± 1.50	0.02*
TAOC (µm Trolox/mg)	1.81 ± 0.75	2.60 ± 0.64	0.03*
OSI (%)	0.79 ± 0.80	0.545 ± 0.37	0.03*

## Discussion

The increasing knowledge about the etiopathogenesis of periodontal disease has led to the emergence of several new therapeutic and preventive strategies for the condition in recent years. Local drug delivery systems deliver the antibiotic to the intended locations, attain a high enough concentration, and last long enough to be beneficial. The current research was done to determine the effectiveness of an antioxidant oral gel (Bluem®) as an adjunct to SRP in Stage II Grade A periodontitis management in terms of clinical and biochemical parameters. 

In this present study, the baseline total antioxidant levels were lower in the periodontitis patients of both groups, which were in line with those of Wei et al. and Baltacioglu et al., and this could be because of the oxidant-antioxidant imbalance during the pathogenesis of periodontal disease [17,18]. Following SRP, both Group 1 and Group 2 patients showed a substantial rise in TAOC and a fall in TOS levels. Akpinar et al. reported that periodontal therapy improved the clinical parameters among smokers as well as non-smokers by lowering TOS and increasing TAOC [19]. Literature evidence highlights the positive effect of non-surgical periodontal treatment by decreasing oxidative stress and increasing antioxidant capacity [20,21]. Chapple et al. suggested that diminished local antioxidant defense may be associated with periodontitis and elevated oxygen radical activity in periodontal inflammation may be reflected in both systemic and salivary levels of TOS [22]. Moreover, Kim et al. observed that salivary TOS decreased immediately following SRP [23].

Furthermore, the present study findings revealed that the antioxidant gel (Bluem®) when used as an adjunct to SRP showed an additive influence among periodontitis patients by decreasing PI, GI, and PD and improving CAL along with the reduction of oxidative stress compared to SRP alone. The current research results cannot be directly compared to those of any other study since this is the first study of its kind to assess the efficacy of Bluem® gel as a local drug delivery agent in terms of assessing oxidant and antioxidant status. However, the results in the current research are indirectly in line with previous studies, where Bluem® gel was used as an adjunct to SRP in periodontitis patients in terms of clinical and microbiological parameters.

Koul et al. assessed the clinical effectiveness and bactericidal properties of both chlorhexidine gel and Bluem® gel in conjunction with non-surgical periodontal therapy (NSPT) in periodontal pockets ≤5 mm [24]. Their findings indicated that both gels were similar and equally efficient in the treatment of chronic periodontitis. When comparing the periodontal index scores and colony-forming units to the baseline values after 30 days, there was a significant decrease. Also, an in vitro study showed that Bluem®, at higher doses, produced a *Porphyromonas gingivalis* inhibitory halo comparable to 0.12% chlorhexidine digluconate [25]. Few other investigations had highlighted that when antioxidant gels were used in conjunction with periodontal treatment, clinical metrics and TAOC were significantly improved, and TOS was reduced [26,27].

Collectively, the present study suggests that Bluem® gel as a local drug delivery agent as an adjunct to SRP showed a greater reduction in GI, PD, CAL, TOS and OSI and improvement in TAOC at the third month follow-up compared to SRP alone. With the above findings, the additive effect of Bluem® gel could be either due to the release of active oxygen or due to the bactericidal effect against gram-negative organisms. However, this study fails to measure the adequate amount of drug, frequency of application, bioavailability, and minimal sample size. Therefore, further randomized controlled trials with larger sample sizes are needed to substantiate these findings. Also, molecular basis of the mode of action of the antioxidant gel needs to be explored in the future.

## Conclusions

From this prospective clinical study conducted over three months, it was found that locally delivered antioxidant gel seems to be effective in reducing oxidative stress and improving the periodontal parameters among stage II grade A periodontitis patients. Traditional SRP with the adjunctive local delivery of antioxidant gel demonstrated an added benefit in improving clinical and biochemical parameters and thereby encouraging the use of the antioxidant gel in clinical practice for the management of periodontitis. However, future randomized controlled trials with larger sample sizes are needed to substantiate these findings. The mode of action of the antioxidant gel and the frequency of administration and its bioavailability need to be explored in the future.
